# Geo-authentic Tibetan medicine: a traditional pharmacological resource for promoting human health and wellness

**DOI:** 10.3389/fphar.2024.1432221

**Published:** 2024-07-23

**Authors:** Ning Wang, Hongkang Zhu

**Affiliations:** ^1^ Department of Food Science and Engineering, Moutai Institute, Renhuai, China; ^2^ School of Food Science and Technology, Jiangnan University, Wuxi, China

**Keywords:** traditional Tibetan medicine, geo-authentic medicinal materials, pharmacology, human health, medicine and food homology

## Abstract

Traditional Tibetan medicine (TTM) is an ancient healing system that has been practiced for more than 2,000 years and involves the use of various medicinal plants for preventing and treating acute mountain sickness, depression, asthma, etc. Geo-authentic medicinal materials, also known as “Daodi herbs” in Chinese, have heightened efficacy and quality relative to their counterparts sourced from alternative geographic locales. In 2024, eight medicinal materials, typified by *Cordyceps sinensis* Sacc., were listed as geo-authentic Tibetan medicine under the administration of the local government. However, there is no comprehensive review on these geo-authentic TTMs, especially with respect to their pharmacological benefits to human health. This review aims to document the pharmacological properties, phytochemical components, safety, toxicity, and future developments of the geo-authentic TTMs that play essential roles in promoting health and wellness. Plant-derived molecules (i.e., polysaccharides, flavonoids, glycosides, terpenoids, and alkaloids) in the TTMs show therapeutic potentials for the management of both mental and physical health. Finally, the applications and prospects of TTM plants are discussed to support the use of these species in folk medicine for human wellness and to promote public health in modern societies.

## Graphical Abstract

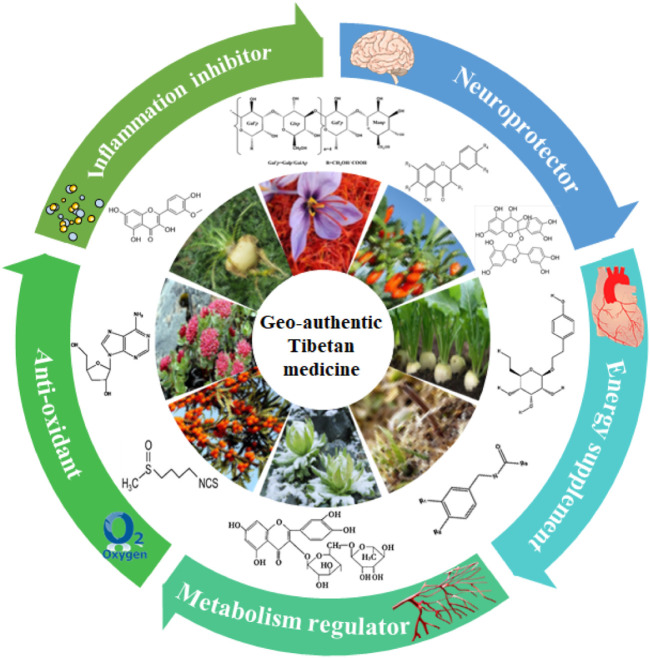

## 1 Introduction

**FIGURE 1:**
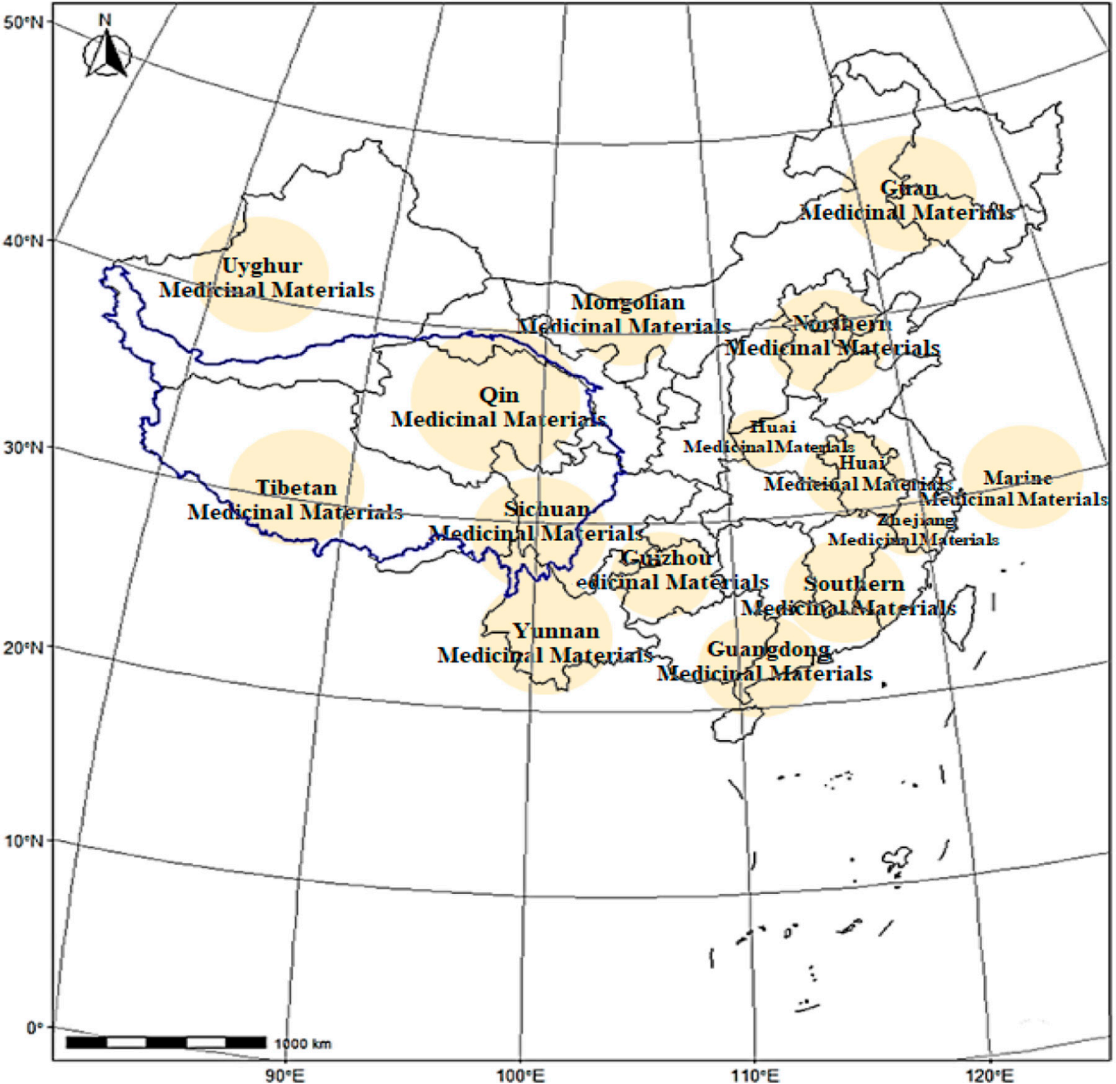
Map of the 15 regions in China known for growing the geo-authentic medicinal herbs.

Geo-authentic medicinal materials, known as “Daodi herbs” in Chinese, are often selected based on extensive use in Chinese medicine clinics; they are cultivated in specific geographic regions and are known for their superior quality, efficacy, and reputation compared to herbs of the same species grown elsewhere (Zhao et al., 2012). This division is based on the natural traits of a topography and geomorphology as well as the essence of the national medical system; this has resulted in the establishment of 15 production regions for geo-authentic medicinal materials in China (Figure 1). The diversity of the biological and medicinal cultures of traditional Tibetan medicines (TTMs) in the Qinghai–Tibet Plateau is amazing; the medicinal materials have been widely used in conventional medicine for thousands of years to treat conditions such as acute mountain sickness, depression, and asthma, among others. Following the prescribed protocols and selection criteria, the Tibetan Pharmaceutical Association has directed its attention toward Tibetan-produced herbs, culminating in the release of the most recent list of eight geo-authentic TTMs in 2024: *Cordyceps sinensis* Sacc*.*, *Rhodiola rosea* L., *Saussurea involucrate*, *Onosma hookeri*, *Dracocephalum tanguticum* M., *Carum carvi* L., *Gymnadenia conopsea* L., and *Brassica rapa* L.

The bioactive components in these plants have been studied extensively for their pharmacological effects, and significant advances have been made in this field. Some of these medicinal plants, like *R. rosea* L., *B. rapa* L., and *S. involucrate*, grow at high altitudes. Most of the aforementioned species are famous for their pharmacological potentials to promote health in traditional culinary and medical practices (Chen, 2023). These medicinal plants have been listed as the top-grade drugs in literature on Tibetan medicine and have been used for tonifying qi and deficiencies for over 3,800 years (Hao et al., 2018). Owing to the renewed interest in the reapplication of traditional medicinal plants in recent times, there have been new investigations on the use of natural plants in anti-hypoxia and anti-fatigue treatments (Zhou and Jiang, 2019). Given the similarities between fatigue and hypoxia (i.e., weakness, bradykinesia, and depression), potential interactions have been proposed and are still being actively debated (Degens et al., 2006; Huang et al., 2022).

The therapeutic potentials of Tibetan medicinal plants have led to promising results in managing hypoxia, fatigue, and related symptoms (Hua et al., 2021b; Zhu et al., 2021; Zhu et al., 2022a). However, based on current understanding, there is a paucity of scholarly literature offering a comprehensive overview of the eight newly acknowledged geo-authentic Tibetan medicinal materials for the treatment of hypoxia, fatigue, and related symptoms. This review seeks to analyze a selection of these geo-authentic medicinal materials as well as several other high-altitude species commonly utilized in TTMs for their efficacy in treating common symptoms such as hypoxia and fatigue (Li et al., 2018a; Zhao et al., 2021b), in addition to conditions like depression, asthma, and acute mountain sickness (Ma et al., 2011). Finally, we list the opportunities for and challenges of harnessing the geo-authentic traditional medicinal plants.

## 2 Pharmacological properties of the geo-authentic Tibetan medicinal plants

### 2.1 *Cordyceps sinensis* Sacc.

**FIGURE 2:**
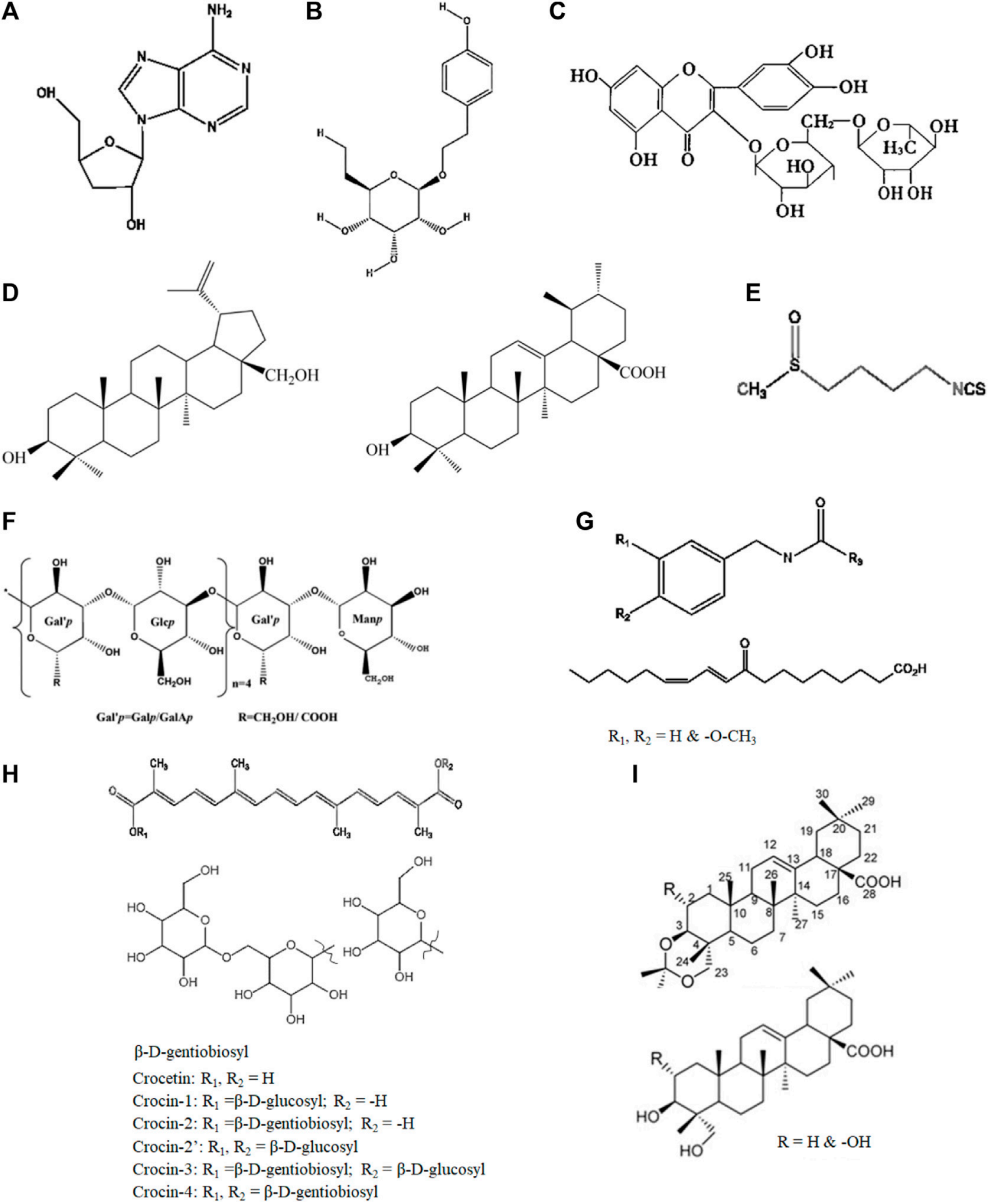
Representative chemical components of the plants used in TTM: **(A)** cordycepin (*Cordyceps sinensis* Sacc.), **(B)** salidroside (*Rhodiola rosea* L.), **(C)** rutin (*Crocus sativus* L.), **(D)** ursolic acid and botulin (*Dracocephalum tanguticum* Maxim.), **(E)** sulforaphane (*Brassica rapa* L.), **(F)** *Lepidium meyenii* Walp. polysaccharides, **(G)** macamides and macaenes (*Lepidium meyenii* Walp.), **(H)** crocin (*Crocus sativus* L.), **(I)** triterpenes (*Stachyurus himalaicus* var. *himalaicus* Hook. f. et Thoms. ex Benth.).

*C. sinensis* Sacc. (3,500–4,500 m) is a type of fungus that is widely used in traditional Chinese medicine (TCM) for its medicinal properties; studies have shown that it can improve oxygen utilization and reduce oxidative stress, which are beneficial for individuals suffering from hypoxia- and fatigue-related disorders (Long et al., 2021b). Cordycepin, which is a nucleoside derivative obtained from *C. sinensis* Sacc. (Figure 2A), has been verified to possess a diverse range of biological activities, including liver protection, antioxidation, immunomodulation, and anti-inflammation (Ashraf et al., 2020). The traditional use of this medicine to improve the body’s hypoxia tolerance and fatigue resistance was recorded as early as the Qing dynasty (1757 AD) (Long et al., 2021a). Additionally, *C. sinensis* has been found to have positive effects on energy metabolism and physical performance, which may also be helpful for individuals experiencing fatigue (Table 1).

**TABLE 1:** Main active ingredients and activities of traditional Tibetan medicinal plants.

No.	Scientific name	Utilized parts	Effective dosage	Safety and oral toxicity	Main active ingredients	Mechanism of pharmacological activity
1	*Cordyceps sinensis Sacc.*	Complex	1.0 g/kg	NOAEL = 2 g/kg	Cordycepin	Increased energy storage, antioxidant (Wang et al., 2003)
2	*Rhodiola rosea* L.	Root rhizome	170 mg/kg	LD_50_ > 10 g/kg	Salidroside, rosavins, and tyrosol	Neuroprotective (Kim et al., 2021; Stojcheva and Quintela, 2022), antioxidant (Xue and Kang, 2020)
3	*Saussurea involucrata* Sch.	Flower	0.15–0.25 g/kg	LD_50_ > 18.75 g/kg	Acacetin, hispidulin, and rutin	Antioxidant (Zheng et al., 1993), anti-fatigue (Lee et al., 2011)
4	*Onosma hookeri*	Root	4.2–6.3 g/kg	—	Polysaccharides and naphthoquinones	Immunostimulant (Wu et al., 2023), anti-inflammatory (Zhou et al., 2021), antioxidant (Wu et al., 2020a)
5	*Carum carvi* L.	Seeds	800–1,600 mg/kg	NOAEL = 3.2 g/kg	Volatile oils, fatty acids, and phenolic acids	Anticonvulsant (Showraki et al., 2016), antioxidant (Kamaleeswari and Nalini, 2006), anti-inflammatory (Keshavarz et al., 2013)
6	*Dracocephalum tanguticum* Maxim.	Whole grass	1–5 g/kg	18.8 ± 0.6 g/kg (intraperitoneal injection) 142.3 ± 4.2 g/kg (intragastric gavage)	Volatile oils, triterpenes, and flavonoids	Antianoxic (Hai et al., 1997), anti-inflammatory (Wang et al., 2024)
7	*Gymnadenia conopsea* (L.) R. Br.	Tuber	1–4 g/kg	LD_50_ > 10 g/kg	Benzylester glucosides, polysaccharides, and alkaloids	Antioxidant (Kanliang, 2016)
8	*Brassica rapa* L.	Root	1.0 g/kg	NOAEL = 18.6 g/kg	Polysaccharides	Removal of accumulated metabolites (Li et al., 2022), antioxidant (Tang et al., 2018), anti-fatigue (Zhu et al., 2022b)
9	*Lepidium meyenii* Walp.	Root	0.1–1 g/kg	LD_50_ > 17 g/kg	Polysaccharides, macaenes, and macamides	Removal of accumulated metabolites, improved energy metabolism (Hongkang et al., 2021)
10	*Crocus sativus* L.	Filament	300 mg/kg	LD_50_ = 4.12 g/kg	Polysaccharides and crocin	Anti-inflammatory and antioxidant (Akbari-Fakhrabadi et al., 2019a)

Abbreviations: LD_50_, median lethal dose; NOAEL, no observed adverse effect level.

### 2.2 *Rhodiola rosea* L.

*R. rosea* L. is considered as the king of Tibetan medicine and grows at an altitude of 1,800–4,000 m; it has been traditionally used in eastern and northern Europe, Asia, and North America (Cui et al., 2014), while the Qinghai–Tibet Plateau is generally considered as the center of its origin and diversity (Brinckmann et al., 2021). The *Rhodiola* species has been extensively used in tonics since time immemorial for its medicinal qualities in traditional folk medicines in China, Tibet, and Mongolia (Cunningham et al., 2020). *R. rosea* L. promotes physical/cognitive vitality as well as recovery from prolonged and minor physical exhaustion (Khanum et al., 2005; Angeli et al., 2018). In addition to the treatment of hypoxia and fatigue, it is applied to treat associated symptoms, such as poor appetite, sleep difficulties, irritability, hypertension, and headaches (Kelly, 2001).

Glycosides are unique secondary metabolites that display rich effects against human diseases and metabolic disorders. The anti-hypoxia and anti-fatigue properties of *Rhodiola* may be related to salidroside (Figure 2B) and flavones. *R. rosea* L. extracts (600 mg/day) containing salidroside (C_14_H_20_O_7_, 2.62%) have been shown to significantly increased exercise capacity and ameliorate exhaustive exercise-induced stress damage. Hou et al. (2020) reported that *Rhodiola* (salidroside, 1.27 mg/mL) ameliorated fatigue via suppression of mitophagy in the skeletal muscles during exhaustive exercise. The protective effects of salidroside against CoCl_2_-simulated hypoxia injury in PC12 cells have also been reported (Tang et al., 2021).

### 2.3 *Saussurea involucrata*

*S. involucrata* (Kar. et Kir.) is a well-known traditional medicinal plant that grows in high mountains (elevation over 4,000 m) covered by snow (Li et al., 2017a). It grows under harsh climatic conditions and takes more than 8 years to mature before harvesting. Owing to its great pharmacological activities, slow growth, and high demand, *S. involucrata* is rare and important in various ethnomedical systems, including TCM, Uyghur, Mongolian, and Kazakhstan medicine (Ma et al., 2014; Han et al., 2016). Since the 1990s, there is increasing attention on the anti-hypoxia and anti-fatigue properties of *S. involucrata*, which can significantly strengthen and invigorate the body, partly owing to its antioxidant capacity (Zheng et al., 1993). Petroleum ether extracts of *S. involucrata* were found to exhibit high neuroprotective effects and reverse damage to the brain caused by acute mountain sickness (Ma et al., 2014; Jing et al., 2016). Both tissue cultures (Jia and Wu, 2008) and ethanolic extracts (Lee et al., 2011) of *S. involucrata* have been shown to significantly enhance the endurance capacities of mice, showing their anti-fatigue activities. In traditional Tibetan/Uyghur medicine, it has long been used under the herbal name of “Tianshan Snow Lotus” for preventing and treating a wide spectrum of disorders. It is famous for its outstanding efficacy in removing dampness, eliminating inflammation, and improving blood circulation and has long been employed to treat acute mountain sickness, fatigue, arthritis, dysmenorrhea, and stomachache (Ma et al., 2011).

Mosloflavon in petroleum extract, such as moslosooflavone, negletein, and 5,6-dihydroxy-7,8-dimethoxyflavone, has been associated with the anti-hypoxia ability of *S. involucrata* (Jing et al., 2016). Dozens of compounds, including flavonoids, phenylpropanoids, and sphingolipids, have also been isolated, characterized, and identified in recent years (Jia et al., 2014; Gong et al., 2020). Rutin (Figure 2C) is a flavonoid and a principal component in *S. involucrata* that can alleviate injuries to the brain (Celik et al., 2020), heart (Diwan et al., 2017), and skeletal muscles (Kappel et al., 2013). A supplement of rutin (60 mg/kg) was shown to significantly attenuate physical fatigue via upregulation of the proliferator-activated receptor-α coactivator and sirtuin 1 expression (Su et al., 2014).

### 2.4 *Onosma hookeri*

The traditional Chinese medicinal herb known as “Zi Cao” has a long history of use as both a medicinal and culinary plant (Wu et al., 2023). However, there is limited documentation on the specific variety of “Zi Cao” sourced from Tibet, specifically *O. hookeri*, despite its classification as a multisource medicinal material. *O. hookeri* is the root of the *O. hookeri* Clarke. var. *longiforum* Duthie and *O. hookeri* CB Clarke. varieties that are predominantly found in the eastern and southern regions of Tibet.

The active compounds isolated from *O. hookeri* include naphthoquinones, alkaloids, flavonoids, and other constituents. Additionally, tigloyl-shikonin, a derivative of shikonin and an acidic polysaccharide with hydrophilic characteristics has been identified in *O. hookeri* Clarke. var. *longiforum* Duthie (Wu et al., 2020b; Wu et al., 2023). The acidic polysaccharide derived from *O. hookeri* Clarke. var. *longiforum* Duthie, which has shown efficacy in boosting immunity *in vitro* and *in vivo*, indicates that the distinct plateau surroundings could influence its metabolic processes and stimulate the synthesis of biologically active substances, ultimately aiding in the identification of polysaccharides with immunomodulatory effects (Wu et al., 2023). Research has also demonstrated that naphthoquinone-enriched ethanolic extracts from the roots of *O. hookeri* Clarke. var*. longiforum* Duthie exhibit strong antioxidant properties, suggesting their potential utility as a natural antioxidants (Wu et al., 2020a).

### 2.5 *Carum carvi* L.

*C. carvi* L. is a perennial herbaceous plant belonging to the Umbelliferae family and primarily thrives in grasslands, ravines, riverbanks, and slopes at altitudes exceeding 4,000 m above sea level. This plant is a significant medicinal resource in Tibetan medicine, with historical records in ancient pharmacopoeias and local chronicles documenting the use of its roots and fruits for medicinal purposes.

The main components of *C. carvi L*. are primarily volatile oils and fatty acids, with minor quantities of flavonoids and phenolic acids (Mahboubi, 2019). The pharmacological properties of *C. carvi* L. encompass antimicrobial (Kocić-Tanackov et al., 2019), antioxidant (Kamaleeswari and Nalini, 2006), and anti-inflammatory (Keshavarz et al., 2013) effects as well as the potential to enhance gastrointestinal function, mitigate obesity, and demonstrate anticonvulsant properties (Lemhadri et al., 2006; Showraki et al., 2016; Krueger et al., 2020), among other therapeutic benefits. The anti-aflatoxigenic, antioxidant, and antimicrobial effects of *C. carvi* L. oil along with its reputation as a spice have been used in industries as natural preservatives and antioxidant agents instead of synthetic substances (Mahboubi, 2019).

### 2.6 *Dracocephalum tanguticum* Maxim.

*D. tanguticum* Maxim. is commonly referred to as “Zhiyangge” in Tibetan medicine and is acknowledged as a prominent medicinal plant in Tibet; it has been incorporated into the standard of Tibetan medicine (Volume 1) and is predominantly distributed in the southeastern region of Tibet, eastern region of Qinghai, southwestern region of Gansu, and western region of Sichuan, as documented by the Editorial Committee of the Flora of China in 1997. *D. tanguticum* Maxim. is a widely used anti-hypoxia medication in Tibetan medicine and is known for its efficacy in enhancing organismal tolerance to hypoxia under both static and locomotor conditions. Studies have shown that this botanical compound can increase blood oxygen levels, elevate ATP concentrations in the brain and heart of hypoxic individuals, and mitigate the depletion of cardiac glycogen reserves. These collective effects contribute to the preservation of normal physiological responses in hypoxic organisms.

At present, the main active components extracted from *D. tanguticum* Maxim. include triterpenes, flavonoids, alkaloids, phenylpropanoids, sesquiterpenoids, and various other compounds within its phytochemical composition (Hongting et al., 2007; Zhang et al., 2007; Liu et al., 2009; Wang et al., 2009; Wang S. Q. et al., 2010; Zeng et al., 2011; Hu et al., 2020; Wang et al., 2024). Hongting et al. (2007) utilized silica gel column chromatography to investigate the fat-soluble chemical constituents of *D. tanguticum* Maxim., resulting in the isolation and identification of ursolic acid, botulin, and additional compounds from its supercritical extract (Figure 2D).

### 2.7 *Gymnadenia conopsea* (L.) R. Br.

*G. conopsea* (L.) R. Br. is a perennial herbaceous flowering plant classified within the family Orchidaceae that exhibits a broad distribution across the temperate and subtropical regions of northern Europe and Asia, spanning altitudes ranging from 200 to 4,700 m (Shang et al., 2017). In mainland China, *G. conopsea* (L.) R. Br. predominantly inhabits western regions, specifically within the provinces of Tibet, Xinjiang, Inner Mongolia, Sichuan, Qinghai, and Gansu (Zi et al., 2008). The botanical species *G. conopsea* (L.) R. Br. is given the vernacular name “Shou Zhang Shen,” which translates to “palm-like ginseng,” owing to the resemblance of its tuber shape to that of a human hand. The pharmacological properties of *G. conopsea* (L.) R. Br. have garnered significant attention in academic research, where studies have confirmed its antioxidant, antiviral, and anti-fatigue characteristics, aligning with its historical medicinal applications. Furthermore, recent investigations have revealed additional pharmacological effects, including immunomodulatory, anti-hyperlipidemic, and neuroprotective actions (Meng et al., 2023). It is noteworthy that the anti-fatigue properties of *G. conopsea* (L.) R. Br. and its derivatives are notably significant in oxygen-deprived environments, making them valuable ingredients in pharmaceuticals, nutraceuticals, and food products.

A total of 170 natural compounds have been identified and characterized from *G. conopsea* (L.) R. Br., all of which were extracted from its tubers. Notably, 54 of these compounds are benzylester glucosides, which are considered as significant constituents. Furthermore, various other compounds, such as dihydrostilbenes, phenanthrenes, polysaccharides, lignans, alkaloids, triterpenoids, flavonoids, steroids, and phenolic compounds, have been detected (Meng et al., 2023).

### 2.8 *Brassica rapa* L.

*B. rapa* L. (also called Tibetan turnip) is native throughout Europe, Russia, Central Asia, and the Near East; it is commonly known as turnip and is one of the oldest cultivated vegetables, with wide consumption in Asia for thousands of years (Cao et al., 2021). It has been widely cultivated for dietary, feeding, and medical purposes owing to its diuretic and detoxifying properties, which can help alleviate water retention and promote elimination of toxins from the body (Yin et al., 2017). The glucosinolates present in this plant have been found to have protective effects against hypoxia-induced oxidative stress and inflammation. Additionally, the high fiber content of *B. rapa* L. can help regulate blood glucose levels and promote healthy digestion, both of which may help reduce fatigue and increase overall energy levels. As an edible plant, *B. rapa* L. is used to achieve anti-hypoxia and anti-fatigue outcomes (Zhao et al., 2021b) as well as for tonic, heat-clearing, detoxification, and neuroprotection applications (Chu et al., 2017; Paul et al., 2019; Hua et al., 2021a).

Polysaccharides are generally recognized as one of the major bioactive substances present mostly in the root parts of plants used in TTM, providing excellent anti-fatigue effects and antioxidant enzyme activities (Li et al., 2018b). *B. rapa* L. polysaccharides (BRP, 94.61% purity) obtained by hot-water extraction with ethanol precipitation have been verified to have anti-fatigue properties (Tang et al., 2018); BRP was shown to effectively extend the swimming time and average swimming speed of mice by regulating the levels of their antioxidative-related enzyme, such as glutathione peroxidase and lactate dehydrogenase at doses of 100 mg/kg bodyweight (bw) per day. Another subspecies called *B. rapa chinensis* (L.) (200 and 400 mg/kg) was observed to have analgesic and antidepressant activities in the Swiss albino mice model (Rahman et al., 2015). Glucosinolates are naturally found in *Brassicaceae* plants, such as *B. rapa* L. (Paul et al., 2019); in addition to polysaccharides, flavonoids, and phenylpropanoids, which normally exist in most edible plants, other active constituents with turnip characteristics such as sulforaphane (Figure 2E) and p-coumaric acid (Li et al., 2018a) have also been shown to have beneficial effects against hypoxia and fatigue. Sulforaphane is one of the most important isothiocyanates and is present in cruciferous vegetables, including *Lepidium meyenii* Walp. and *B. rapa* L.; at a dose of 25 mg/kg, it was shown to upregulate the nuclear factor (erythroid derived 2)-like 2 (Nrf2) and downstream genes, significantly attenuating muscle fatigue and improving exercise performance (Oh et al., 2017). Ikeuchi et al. (2009) found that benzylglucosinolate (0.015 or 0.030 mg/kg) significantly increased mice endurance exercise capacity due to greater utilization of fatty acids as the energy sources.

### 2.9 Other traditional Tibetan medicinal plants and bioactive components

*L, meyenii* Walp. (also called Maca) has been utilized for almost 2,000 years across the Peruvian highlands, South American Andes, Tibetan Plateau, and Taipei Plateau (Gonzales, 2012); it has been traditionally employed in the Andean region for its activities against hypoxia- and fatigue-associated symptoms in the highlands, especially those related to sexual behaviors (Cicero et al., 2001). Daily consumption of spray-dried extracts of red and black *L. meyenii* Walp. (3 g) was shown to improve health-related quality of life (sexual desire, mood, and energy) at low altitudes as well as chronic mountain sickness (CMS) (glycemia, blood pressure, and hemoglobin levels) at high altitudes (Gonzales, 2012). A cross-sectional study suggested that *L. meyenii* Walp. significantly decreased the testosterone/estradiol ratio and IL-6 levels along with low CMS scores (Gonzales et al., 2013). Li et al. (2017b) isolated and purified two fractions of *L. meyenii* Walp. polysaccharides from its aqueous extract, which presented dose-dependent positive effects on forced swimming in mice. Other *L. meyenii* Walp. polysaccharides were isolated from a water extract of *L. meyenii* Walp. tubers, demonstrating significant anti-fatigue effects (Figure 2F) (Weimin et al., 2017). Remarkably, several studies showed that macamides (Figure 2G), which are a series of characteristic compounds in the root of *L. meyenii* Walp., had significant anti-fatigue activities (Zhu et al., 2020).

In addition to the aforementioned plants, several others, such as *Nitraria tangutorum* Bobrov. (1,900–3,500 m) (Suo et al., 2005), *Hordeum vulgare* L. (4,200–4,500 m) (Kim et al., 2012), and *Crocus sativus* L., have also been shown to have different degrees of anti-hypoxia and anti-fatigue activities (Table 1). Interestingly, most of these species having considerable anti-hypoxia and anti-fatigue properties grow in plateau regions under extreme cold climates. They have adjusted growths and stresses to adapt to the high altitudes and frigid weather conditions (Reyes et al., 2020). *C. sativus* L. contains several bioactive compounds, including crocin (Figure 2H), crocetin, and safranal (Akbari et al., 2018), which are responsible for its pharmacological effects, including antioxidant, anti-inflammatory, and neuroprotective properties. Terpenoids are one of the largest classes of chemical components in natural products; they are rich resources for developing pharmaceuticals, fragrances, and biofuels. Additionally, triterpenoids have been identified from *Stachyurus himalaicus* var. *himalaicus* Hook. f. et Thoms. ex Benth. (Figure 2I) (Wang Y. S. et al., 2010).

In conclusion, biological multilevel theory describes that plant pharmacology mechanisms may be linked to the habitats of high-altitude medical plants for quantification of the active ingredients and human internal biological processes (Li et al., 2020). The manner in which the plateau environment and climate participate in such regulation of the metabolisms of high-altitude plants and their effects on the pharmacological properties, especially for anti-hypoxia and anti-fatigue activities, may be interesting questions for further exploration.

## 3 Characteristic ethnomedical uses in modern healthcare practices

Given their specific environmental conditions, the majority of TTMs serve distinct and individualized functions in combating hypoxia and mitigating fatigue (Hua et al., 2021b; Zhu et al., 2021). Additionally, a strong correlation exists between hypoxia and fatigue. Hypoxia adversely affects cellular aerobic respiration, leading to tissue hypoxia and the onset of fatigue (Wirth and Scheibenbogen, 2020). The capacity to withstand hypoxia aids in upholding regular energy metabolism, consequently diminishing fatigue. Hypoxia triggers a cascade of metabolic dysfunctions, and the potential anti-fatigue mechanisms may involve management of these metabolic irregularities to sustain homeostasis (Zhu et al., 2022a).

### 3.1 Highlands: focus on hypoxia

Hypoxia or fatigue remains one of the major risk factors for human life and health in the highlands, especially in people with underlying conditions. There are approximately over 140 million people who live above altitudes of 2,500 m across the world, and the highest population density is located above 3,500 m (Chu et al., 2017). In recent years, there have also been over 40 million visitors from plains who have been exposed to plateau environments yearly through traveling, altitude training, or military needs, who could be plagued by high-altitude hypoxia and related illnesses (Pena et al., 2022). The Tibetan Plateau, with an average altitude exceeding 4,000 m, is one of the highest regions on Earth (Dong et al., 2020). Residents of the Tibetan Plateau have often suffered the harsh highland environment from birth for thousands of years, thus necessitating the development of TTMs to combat hypoxia, fatigue, and related symptoms (Chu et al., 2017).

Alpine plants have demonstrated multiple pharmacological activities for regulating acute mountain sickness and physical fatigue (Khanum et al., 2005; Larmo et al., 2008; Li et al., 2017b; Akbari-Fakhrabadi et al., 2019a). Most of these high-altitude plants have been used in TCM for over 2,000 years. Interestingly, these medicinal plants, which are commonly used to replenish the kidney and spleen as well as soothe the lung (Chinese Pharmacopoeia Commission, 2010), have long been used to ameliorate human conditions associated with hypoxia, fatigue, and related symptoms (Ma et al., 2011; Qian et al., 2011; Gonzales et al., 2013; Long et al., 2021b; Cao et al., 2021). It is fascinating and awe-inspiring for permanent residents and tourists to live in the highlands, but symptoms of altitude sickness can be unbearable given the low atmospheric pressures and low oxygen levels (León-Velarde et al., 2005). Thin air in the highlands may cause acute and chronic hypoxia, inducing increased right ventricular afterload and pulmonary hypertension (Grimminger et al., 2017). Fortunately, most native foods and traditional medicines in such regions may potentially provide protection to highland dwellers for multisystem regulation (Zhu et al., 2022a). For example, *R. rosea* L. is widely used by highland inhabitants to ameliorate depression, altitude sickness, and fatigue (Panossian et al., 2010). By enhancing immunity and hypoxia tolerance, these products combine healthcare properties with medicinal value. Thus, these nutraceuticals can be widely used in the highlands to prevent and treat symptoms of altitude hypoxia (Hou et al., 2021).

In humans, muscle fatigue is often defined as an exercise-induced decrease in producing force that relies on contractile mechanisms. On the other hand, impaired circulation or insufficient oxygen supply experienced in varying degrees by patients with chronic fatigue syndrome (CFS) can lead to tissue hypoxia as well as clinical manifestations such as fatigue and dizziness (Wirth and Scheibenbogen, 2020). Therefore, hypoxia-related genes like the hypoxia-inducible factors (HIFs) have been proven to be appropriate targets for fatigue resistance and exercise tolerance via hypoxic adaptation (Dang and Gao, 2008), which is a commonly suggested treatment for fatigue-related disorders in several physiologic and pathologic processes (Halley et al., 2019; Holland et al., 2020). Two ancient Tibetan pharmacopoeias, namely *The Four Medical Tantras* and *Jing Zhu Materia Medica*, state that *B. rapa* L. has effects like “tonic and anti-hypoxia,” “alleviating fatigue,” and “heat-clearing and detoxification,” and has been traditionally exploited for alleviation of plateau hypoxia, fatigue, and associated symptoms (Chu et al., 2017). Chu et al. (2017) also found that consumption a powder made of Tibetan turnips (7.5 g twice daily) for a week could improve oxygen uptake and delivery, body antioxidant capacity, and cardio-pulmonary conditions in healthy humans, based on the Bruce treadmill protocol. A 7-day human self-control and single-blind human feeding trial showed that *B. rapa* L. improved hypoxia tolerance in healthy humans, characterized by improved pulse oxygen saturation (SpO_2_) (Chu et al., 2017). Chu et al. (2018) found that *B. rapa* L. improved the tolerances of athletes to hypoxia, which might be attributed to enhancements of their cardiopulmonary functions and oxygen metabolisms. Because of the bioactivities of anti-hypoxia medications, they can be used in cerebral stroke as inhibitors of reactive oxygen species (ROS) induced by oxygen glucose deprivation/reperfusion (OGD/R) to restore mitochondrial expression via the PI3K/Akt/mTOR pathway (Hua et al., 2021b).

### 3.2 Flatlands: focus on fatigue

Fatigue is best described as an overwhelming feeling of tiredness and exhaustion; similar to the symptoms of hypoxia, it is characterized by several processes like angiogenesis and energy metabolism disorders (Wang et al., 2019). In general, fatigue can be classified as central or peripheral fatigue at different levels of the motor pathway. The former is commonly caused by changes in the central neurotransmitters and is perceived in the central nervous system (CNS), which can lead to severe neurological damage and dysfunction by hypoxia (Hanyi et al., 2021). It can be accompanied by multiple mental disorders, such as psychological loss of motivation or appetite, sleepiness, and reduced psychological alertness (Kwak et al., 2020). As a progressive, exercise-induced degradation of muscle voluntary activation, it is closely related to peripheral fatigue (Hogan et al., 2020). Specifically, energy consumption through glycogen, adenosine triphosphate (ATP), and metabolic factors like H^+^, lactate, Pi, and ROS generated by strenuous exercise can influence the contractile functions of skeletal muscles to accelerate fatigue (Wu et al., 2014). In Tibetan medicine, *R. rosea* L. has been used to relieve high-altitude sickness, anoxia, and mountain malhypoxia (Qian et al., 2011). *R. rosea* L. has been categorized as an adaptogen by Russian researchers for its pharmacological activity of preventing chemical, biological, and physical stressors. Several anti-fatigue trials have identified multiple pharmacological effects of *R. rosea* L. extracts (Hung et al., 2011). Ballmann et al. (2019) found that short-term supplementation with *R. rosea* L. (golden root extract, 1,500 mg/person) enhanced the anaerobic exercise performances of college-age females. Another clinical trial on healthy men showed that chronic *R. rosea* L. supplementation contributed to better mental and physical performances, with enhanced total antioxidant capacity (Jowko et al., 2018).

Although the partial pressure of oxygen is normal in the flatlands, the rich phytochemical profiles of high-altitude plants have been exploited further with high commercial value (Zhu et al., 2022a). Based on their ethnomedical uses and market demands, a series of products based on the value chain of high-altitude medicinal plants are being developed rapidly. Nowadays, *R. rosea* L. has been shown to have medicinal and food homology (El-Houri et al., 2014), and *L. meyenii* Walp., which is a typical example of a superfood, has been certified as a new food resource in China (Zhu et al., 2020). *L. meyenii* Walp. rose to fame in the last decade for its reputation as “plant Viagra” mainly because of its fertility-related activities, which were shown to improve sexual behaviors (Cicero et al., 2001), litter size (Ruiz-Luna et al., 2005), and spermatogenesis quality (Gonzales et al., 2006). Based on the pharmacological characteristics of *S. involucrata*, many snow lotus products like cosmeceuticals, food supplements, and medications are now available in the current herbal market. Thus far, international exports of high-altitude plant products in China, mostly from the Qinghai–Tibet Plateau, have become a major driver of commercial trade. For example, *R. rosea* L. has been formulated into capsules to achieve significant improvements in maximal oxygen uptake (VO_2max_) and endurance performance among healthy males after dietary supplementation for 7 weeks (Zhang et al., 2009). Medicinal herbs are often used as nutritious and functional foods nowadays, and high-altitude plants have been utilized for several health-associated functions, such as anti-fatigue (Li et al., 2017b), antioxidant (Lee and Chang, 2019), neuroprotective (Yu et al., 2019), prebiotic, and anti-inflammatory (Lee et al., 2020) properties, gradually garnering attention as well as increased consumption worldwide over the past 20 years.

## 4 Safety and toxicity

Most of the compounds present in high-altitude plants are orally administered, with low or no toxicity. The oral median lethal dose (LD_50_) of *Rhodiola* root aqueous extract was observed to be more than 10 g/kg (Gupta et al., 2008), and the LD_50_ of the *L. meyenii* Walp. ethanolic extract was greater than 2 g/kg (Abarikwu et al., 2020). It was also observed that a single dose of *L. meyenii* Walp. extract at 17 g/kg did not result in acute toxicity in rats (Chen et al., 2021), suggesting that the concentrations required for exhibiting bioactivities are lower than their toxic concentrations (Hudson et al., 2018). The acute toxicity profiles of *C. carvi* L. suggests that the highest non-lethal doses of parsley essential oil and aqueous extract are 400 mg/kg and 3,200 mg/kg, respectively (Showraki et al., 2016). Furthermore, it was found that the LD_50_ value of *C. sativus* L. in mice was 4,120 ± 556 mg/kg (Bahmani et al., 2014). Mice and rats were subjected to different doses (1.00, 2.15, 4.64, and 10 g/kg) of *G. conopsea* (L.) R. Br. tubers, and no statistically significant changes in their behaviors and growths were observed compared to the control group over a 2-week observation period, suggesting that the LD_50_ of *G. conopsea* (L.) R. Br. tubers exceeds 10 g/kg (Dian-Qing and Ying, 2007). An acute toxicity experiment of *D. tanguticum* Maxim. conducted on mice indicated that the LD_50_ values for intraperitoneal injection and intragastric administration were 18.8 ± 0.6 g/kg and 142.3 ± 4.2 g/kg, respectively (Hai et al., 1995). Moreover, research has shown that laboratory-cultured mycelia powder of *C. sinensis* Sacc. is safe and non-toxic for dosages up to 2 g/kg (Meena et al., 2013). No fatalities were recorded in mice administered 18.6 g/kg of *B. rapa* L. polysaccharides (Bakasi et al., 2019).

These scientific studies and centuries of traditional use confirm the acceptability and safety of consumption of high-altitude medicinal products, along with improvements to the mood, energy, and health status, as well as reduced CMS scores (Gonzales-Arimborgo et al., 2016). However, *S. involucrata* is prohibited for use by pregnant women in the Pharmacopoeia of China (Chinese Pharmacopoeia Commission, 2010) owing to its side effect of abortion at 0.3 mL of intravenous administration; in addition, *S. involucrata* has been associated with some nervous and cardiovascular disorders, such as arrhythmia, hypotension, and nausea. Contradictorily, *S. involucrata* injection (Chik et al., 2015) and its culture (Han et al., 2021) have been proven to have no distinct toxicity or adverse effects (Table 1).

## 5 Challenges and prospects

### 5.1 Globalization and modernization research on TTMs

Future developments in TTM will likely involve a combination of traditional knowledge and modern scientific approaches (Luo, 2015). Globalization and modernization have become some of the principal targets of most traditional medicine systems, particularly the herbal drugs and prescriptions of Asia, south/west Africa, and Latin America. However, modernization of TTM is still a long way off owing to its complex ingredients and uncertain mechanisms (Yan et al., 2021). Thus, one of the key segments in the modernization research on TTM is clarification of the chemical compositions and transformation of pharmacological research findings on traditional Tibetan plants into products of commercial value. However, the Tibetan medicine industry is plagued by insufficient and overdrawn Tibetan medicinal resources as well as small-scale enterprises, which poses problems on sustainable and rapid developments (Luo, 2015).

### 5.2 Sustainable development of places growing Tibetan medicinal plants

Places where geo-authentic medicines are produced are termed as “trueborn areas,” and medicinal herbs from trueborn areas are most remarkable in terms of both quality and efficacy, particularly those that grow in the highlands (Tang et al., 2017). However, most of the high-altitude medicinal plants are grown and described with respect to plateau areas, where both economy and productivity are relatively poor. In addition, excessive exploitation of high-altitude plants has resulted in serious regional damage to natural niches owing to the fragile ecological environments on plateaus. Growing and developing high-altitude plants also entails transportation barriers stemming from distance and the lack of intelligence and informatization in the mountains. Based on TCM theory, *L. meyenii* Walp. has been used as a classic medicinal plant for warming and invigorating the kidney (Yang et al., 2018) as well as for spleen-deficiency syndrome (Fei et al., 2020). Compared with people residing at sea levels, delayed healing rates are observed among wounded dwellers at high altitudes as well as increased wound widths; red *L. meyenii* Walp. has been shown to promote skin wound healing at both sea level and high altitudes (Nunez et al., 2017). Thus, sustainable development of medicinal origin places and wild collection standards must be prioritized over the next several decades (Brinckmann et al., 2021).

### 5.3 Adulteration in the Tibetan medicinal materials market

Traditional Tibetan medicinal plants are critically endangered given the increasingly lucrative market for TTM in recent times, especially the “Daodi herbs”. The present discriminations between raw and processed high-altitude medicines are mostly focused on important active components, such as salidroside in *R. rosea* L. and macamides in *L. meyenii* Walp. However, there is increasing concern about economically motivated adulteration (EMA) and substitution of inferior species admixtures in the raw high-altitude herbal trade, which can harm consumer faith (Srirama et al., 2017). It is also particularly alarming that the reported adulterants in medicinal plants may have uncertain effects on its safety and efficacy. Thus, it is imperative to enforce universally acceptable pharmacopoeia-specified standards on products to authenticate quality control (QC) of Tibetan medicinal plants.

## 6 Conclusion

TTM is homologous to the roots of the Chinese traditional systems of medicine, where the concept of homology of medicine and food is a fundamental aspect. Adhering to this holistic therapeutic philosophy, TTM is based on herbal medicines that have natural synergistic effects (multiple compounds with multiple targets and one pathway), emphasizing the essential role of food and herbs in promoting health and preventing illness. In particular, geo-authentic Tibetan medicines carefully chosen through extensive clinical experience by practitioners of TCM exhibit superior quality and therapeutic effectiveness compared to similar herbs from other regions, thus enjoying widespread popularity. In addition, compared to mainstream allopathic medicines, it is generally known natural plants, especially those that are homologous medicine and food, are considered to be safer with fewer side-effects.

The market for geo-authentic Tibetan medicines is witnessing an ever-increasing demand globally as herbal medicines are being preferred by increasing numbers of elderly and health-conscious people. Traditional medicinal plants tend to be potential therapeutic strategies for more tolerant to some chronic disorders in daily life, which may be critical for improving the physical states in subhealth populations. Although further research is necessary to fully understand the mechanisms of action and potential benefits of TTMs, their use in folk medicine serves as a valuable starting point for modern medical research.
